# Multidisciplinary approach to Gorlin-Goltz syndrome: from diagnosis to surgical treatment of jawbones

**DOI:** 10.1186/s40902-022-00355-5

**Published:** 2022-07-18

**Authors:** Francesco Spadari, Federica Pulicari, Matteo Pellegrini, Andrea Scribante, Umberto Garagiola

**Affiliations:** 1 Department of Biomedical Surgical and Dental Sciences, Maxillo-Facial and Odontostomatology Unit, School of Orthodontics, Fondazione IRCCS Ca’ Granda Ospedale Maggiore Policlinico, University of Milan, Milan, Italy; 2 Department of Clinical-Surgical, Diagnostic and Pediatric Sciences Section of Dentistry, University of Pavia, Pavia, Italy

**Keywords:** Gorlin-Goltz syndrome, Protein patched homolog 1, Basal cell carcinoma, Odontogenic keratocysts, Palm-plantar pits, “En bloc” resection, Enucleation, Marsupialization

## Abstract

**Background:**

Gorlin syndrome, also known as Gorlin-Goltz syndrome (GGS) or basal cell nevus syndrome (BCNS) or nevoid basal cell carcinoma syndrome (NBCCS), is an autosomal dominant familial cancer syndrome. It is characterized by the presence of numerous basal cell carcinomas (BCCs), along with skeletal, ophthalmic, and neurological abnormalities. It is essential to anticipate the diagnosis by identifying the pathology through the available diagnostic tests, clinical signs, and radiological manifestations, setting up an adequate treatment plan.

**Main body:**

In the first part, we searched recent databases including MEDLINE (PubMed), Embase, and the Cochrane Library by analyzing the etiopathogenesis of the disease, identifying the genetic alterations underlying them. Subsequently, we defined what are, to date, the major and minor clinical diagnostic criteria, the possible genetic tests to be performed, and the pathologies with which to perform differential diagnosis. The radiological investigations were reviewed based on the most recent literature, and in the second part, we performed a review regarding the existing jawbone protocols, treating simple enucleation, enucleation with bone curettage in association or not with topical use of cytotoxic chemicals, and “en bloc” resection followed by possible bone reconstruction, marsupialization, decompression, and cryotherapy.

**Conclusion:**

To promote the most efficient and accurate management of GGS, this article summarizes the clinical features of the disease, pathogenesis, diagnostic criteria, differential diagnosis, and surgical protocols. To arrive at an early diagnosis of the syndrome, it would be advisable to perform radiographic and clinical examinations from the young age of the patient. The management of the patient with GGS requires a multidisciplinary approach ensuring an adequate quality of life and effective treatment of symptoms.

## Background

Gorlin syndrome, also known as Gorlin-Goltz syndrome (GGS) or basal cell nevus syndrome (BCNS) or nevoid basal cell carcinoma syndrome (NBCCS), is an autosomal dominant familial cancer syndrome. It is characterized by the presence of numerous basal cell carcinomas (BCCs), along with skeletal, ophthalmic, and neurological abnormalities [1–4].

Gorlin-Goltz syndrome is caused by mutations in the protein patched homolog 1 (PTCH1) gene that codes for a transmembrane receptor, which recognizes the sonic hedgehog (SHH) signaling protein. There is a high penetrance, that is, the frequency with which an allele occurs phenotypically within a population, with variable expressiveness. De novo mutations account for approximately 20–30% of BCNS cases [5].

The prevalence of Gorlin syndrome is between 1:57,000 and 1:256,000 individuals [6], and in Italy, the prevalence is 1:256,000 [7]; the incidence at birth was confirmed to be 1:18,976 cases [8]. The disease affects men and women in an even distribution (1:1.3). Although the disease affects all ethnicities, African Americans and Asians represent only 5% of cases and it is more often diagnosed accidentally in the presence of extracutaneous signs such as odontogenic keratocysts (OKC) than BCCs [5].

The purpose of this literature review is to identify the pathology through available diagnostic tests, clinical signs, and radiological manifestations and set up an appropriate treatment plan to anticipate diagnosis. The review also analyzes the surgical aspect of the treatment of the typical lesions of the oral cavity that appear in these patients, the keratocysts, highlighting for each treatment the relapse rate that emerged from the studies conducted.

Clinical features including the etiopathogenesis of GGS are reviewed, and appropriate management with accurate diagnosis, differential diagnosis, radiological investigations, and consolidated surgical protocols is summarized.

## Main text

### Part 1 — Etiopathogenesis, diagnosis, and radiological investigations

#### Etiopathogenesis

Gorlin syndrome is caused by a mutation in PTCH1, a tumor suppressor gene located on chromosome 9q22.32.

The PTCH gene encodes a transmembrane receptor protein that recognizes the Hedgehog (Hh) protein, encoded by SHH, the gene found on chromosome 7q36.3. This signal transduction pathway is called the Hedgehog signaling pathway, which is an evolutionary pathway of signal transduction involved in over 50% of forms of cancer. Mammals have three homologues of Hedgehog called the Desert Hedgehog (DHH), the Indian Hedgehog (IHH), and the Sonic Hedgehog (SHH), which is the most studied. This internal signaling pathway takes its name from a polypeptide ligand, that is, the Hh protein, presented in fruit flies of the genus *Drosophila*: it is claimed that fruit fly larvae without the Hh gene resemble hedgehogs.

**Fig. 1:**
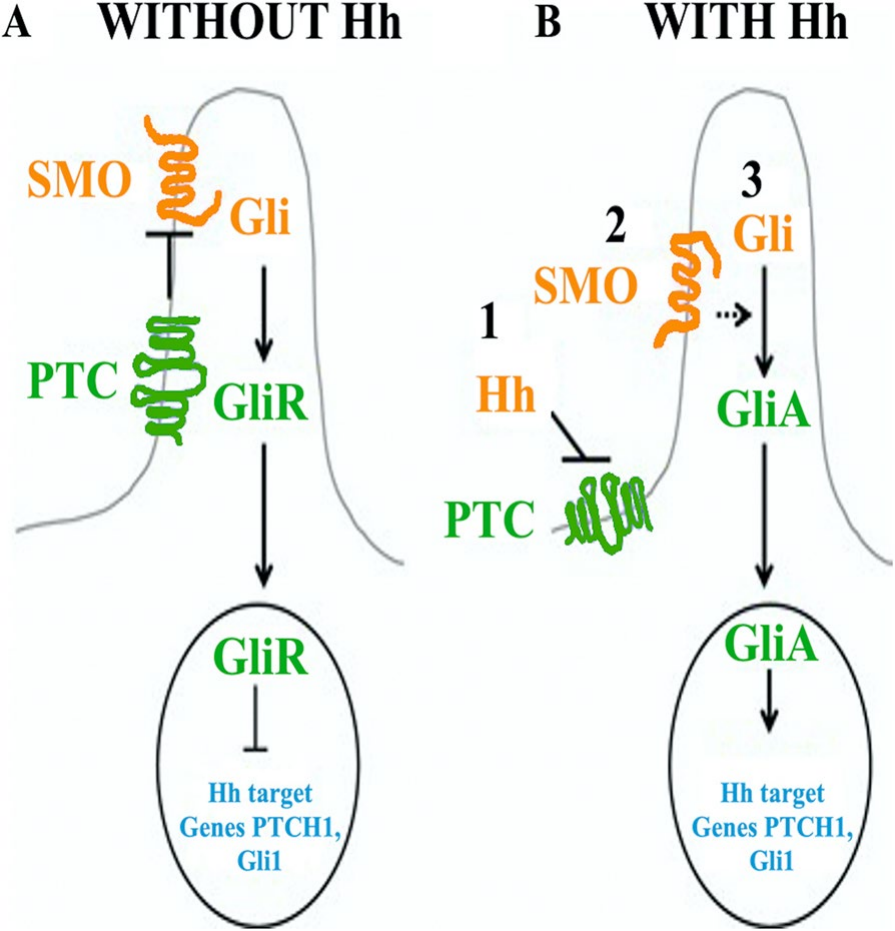
SHH signaling pathway. Hh receptor PTC inhibits SMO signaling via an unknown mechanism, in the absence of the Hh ligands, and Gli molecules, processed into repressor forms, turn off the Hh-signaling pathway (**a**). PTC is unable to inhibit SMO in the presence of Hh. Gli molecules, processed to active forms (GliA), activate Hh target genes (**b**). Abbreviations: Gli glioma-associated oncogene, Gli1 glioma-associated oncogene 1, GliA glioma-associated oncogene activator, GliR glioma-associated oncogene repressor, Hh hedgehog, SMO smoothened, PTC patched, PTCH1 protein patched homolog 1

As shown in Fig. 1, when SHH reaches its target cell, it binds to the patched-1 receptor (PTCH1) [9]. In the absence of such a ligand, PTCH1 inhibits Smoothened (SMO), a downstream protein in the pathway. It has been suggested that SMO is regulated by a small protein, whose intracellular localization is controlled by PTCH. PTCH1 has a sterol binding domain called sterol-sensing domain (SSD), which has proved essential for the suppression of SMO activity. A new theory claims that PTCH regulates SMO by acting as a sterol pump, removing the oxysterols created by 7-dehydrocholesterol reductase. Following the link between SHH and PTCH1 or in the presence of a mutation in the SSD domain of PTCH1, this pump is deactivated allowing the oxysterols to accumulate around SMO. This accumulation allows SMO to activate or remain on the membrane for a long time, leading to the activation of the nuclear transcription factors glioma-associated oncogene homolog GLI1 and GLI2, activators, and GLI3, repressor. The activated GLI accumulates in the nucleus controlling the transcription of target genes [10–13].

In addition to PTCH1, mammals have another hedgehog receptor namely PTCH2, whose gene sequence is in common with PTCH1 at 54% and binds SHH. However, PTCH2 is the most expressed in the testis and mediates DHH signaling. In the absence of binding to the ligand, PTCH2 has a reduced ability to inhibit SMO and furthermore its overexpression does not replace PTCH1 mutated in BCC [14–16].

This pathway plays a fundamental role in cell growth and differentiation, in the regulation of organogenesis in vertebrates, in the differentiation of the fingers, and in the development of the brain and spinal cord, eyes, and teeth. Indeed, the SHH protein is necessary for the development of the anterior part of the brain (forebrain), participating in establishing the midline for the lower part (ventral surface) of the forebrain; moreover, together with other signaling proteins, it is necessary to form the cerebral hemispheres. SHH also plays an important role in eye formation: During early development, the eye cells form a single structure called ocular field. This structure is in the center of the primary face. The sonic hedgehog signaling pathway leads the ocular field to separate into two distinct eyes [17–24].

Homozygous inactivation of the PTCH gene leads to cancerogenesis and the formation of multiple BCCs and other neoplasms. Patients with Gorlin syndrome inherit a defective copy of the tumor suppressor gene (first stroke) and may acquire a mutation in the second healthy tumor suppressor, caused for example, by ultraviolet light or ionizing radiation (second stroke).

Recently, gene alterations have been identified in the suppressor of fused homolog (SUFU) gene, present on chromosome 10q24.32, and in the PTCH2 gene, located on chromosome 1p34.1, in patients with Gorlin syndrome. Gene alterations in the PTCH2 gene may not be conclusive [25].

Gene alterations in the SUFU gene cause functional deficits in the cytoplasmic protein SUFU. This is an important inhibitor of the GLI1 protein, also known as glioma-associated oncogene, encoded by the GLI1 gene. The SUFU protein binds to the GLI1 protein sequestrating it in the cytoplasm, thus preventing its endonuclear translocation and, consequently, its activity as a transcriptional inducer. Mutations in the SUFU gene lead to the formation of a non-functional protein leading to the loss of one of the most important mechanisms that limit Hedgehog signaling. Patients with SUFU mutations have a 20-fold higher risk of developing medulloblastoma (33%) than patients with PTCH1 mutations in Gorlin syndrome (<2%) [26–28].

#### Diagnosis

Diagnosis of BCNS requires the presence of two major or one major and two minor clinical criteria [5, 7, 29–32]. Major criteria include:

- Multiple BCCs (> 2) or 1 BCC within ≤ 20 years of age. They are present in 80% of affected patients and the number can vary from a few tens to hundreds, with a high clinical and histopathological heterogeneity. They arise on the face, back, and chest and are usually not aggressive but in the absence of treatment they can manifest a certain degree of local invasiveness. Primary prevention measures need to be implemented to reduce the risk of developing BCC in dark-skinned individuals and light-skinned individuals living in countries with a warm, sunny climate; patients with phototype 1 (Celtic type, i.e., light skin, blue eyes, freckles, and red or blond hair) and, among them, patients with the altered melanocortin 1 receptor gene (MC1R, variant rs1805007). The age of onset of the syndrome could change
- Jawbone odontogenic keratocyts diagnosed histologically; in orthopantomography, it is identified as an area of translucency
- Palm-plantar pits (≥2), present in 87% of patients. They are asymmetrical dimples having a diameter of 2–3 mm and a depth of 1–3 mm. They are more evident when hands and feet are immersed in warm water for 10 min. The dimples may appear as white “punch” lesions or as pink “pinheads.” They usually occur in the second decade of life and are caused by a partial or complete absence of the cutaneous keratinized layer in the palmar and plantar regions.
- Lamellar calcification of the falx cerebri or clear evidence of calcification shown under the age of 20 years. Calcification of the falx cerebri is usually present and is visible on the anteroposterior radiograph of the skull
- Bifid/fused/spread ribs
- First-degree relative with BCNS

Minor criteria include:

- Medulloblastoma
- Lympho-mesenteric or pleural cysts
- Facial dysmorphisms: cleft lip/palate, macrocephaly, orbitofrontal cortex (OFC), greater than 97th percentile), rounded forehead, coarse facial appearance and facial milia
- Ocular anomalies: cataract, coloboma, microphthalmos
- Skeletal anomalies: Sprengel’s deformity (or congenital elevation of the scapula), marked pectus deformity, marked syndactyly and polydactyly
- Radiological anomalies: calcification of the sella turcica, hemivertebrae, fusion or elongation of the vertebral bodies, cuneiform vertebrae, modeling defects of the hands and feet, flame-shaped radiolucency in the hands or feet
- Ovarian and cardiac fibromas

Genetic testing for PTCH1 is suggested for the following situations:

1. Confirmation of diagnosis in patients lacking sufficient clinical diagnostic criteria
2. Predictive testing for at-risk patients with an affected family member but who do not meet the clinical criteria
3. Prenatal testing in the presence of a known familial mutation

Approaches to molecular testing may include serial testing of a single gene, the use of a multigene panel, and more comprehensive genomic testing [33–39].

The suggested order for performing a serial test of a single gene is:

1. Analysis of the PTCH1 sequence
2. Deletion/duplication analysis targeting the PTCH1 gene
3. SUFU sequence analysis
4. Deletion/duplication analysis targeting the SUFU gene
5. Analysis of PTCH1 ribonucleic acid (RNA)

SUFU molecular testing should be performed first in families with medulloblastoma and without maxillary keratocysts [39].

The execution of a multigene panel that includes PTCH1, SUFU, and other genes of interest may be considered (Table 1) [32].

**Table 1:** Molecular genetic tests used in Gorlin-Goltz syndrome

Gene	Genetic test	Proportion of probands with a pathogenic variant detectable by genetic testing
PTCH1	Gene sequence analysis	50–85%
	Gene deletion/duplication analysis	6–21%
SUFU	Gene sequence analysis	5%
	Gene deletion/duplication analysis	~1%

When considering NBCCS, a tailor-made panel of PTCH1 and SUFU only should be considered optimal as large multigene panels may have reduced sensitivity and may not include gene targeted deletion/duplication analysis or gene analysis. PTCH1 RNA required to identify large rearrangements [33, 39].

More comprehensive genomic testing (if available), including exome sequencing, the portion of the genome capable of encoding a protein, and genome sequencing, may be considered. These tests may provide or suggest a previously not considered diagnosis (for example, mutation of one or more different genes resulting in a similar clinical presentation).

A recent review of 182 genotyped individuals having NBCCS found that individuals with PTCH1 gene mutations were more likely to be diagnosed early (*p* = 0.02), had mandibular keratocysts (*p* = 0.002), and had bifid ribs (*p* = 0.003) or any skeletal abnormality (*p* = 0.003) compared to individuals with no pathogenic variant identified [35]. Approximately 90% of individuals with PTCH1-related NBCCS develop multiple keratocysts of the jaw, and approximately 60% of individuals with a pathogenic variant of PTCH1 have a recognizable appearance with domed forehead, coarse facial features, and milia facial features [32, 35]. The risk of medulloblastoma in PTCH1-related NBCCS was less than 2% [40].

SUFU-related NBCCS is associated with a high risk of medulloblastoma up to 33% (3/9) and a high risk of post-radiation meningioma. Overall, clinical features are milder in individuals with SUFU-related NBCCS, in whom BCCs are less present and no odontogenic keratocysts have been reported [32, 35].

One study reported heterozygous pathogenic missense variants of PTCH1 in five out of 100 unrelated probands with holoprosencephaly. The authors hypothesized that pathogenic missense variants would lead to greater inhibition of PTCH1 on the sonic hedgehog signaling pathway, unlike the mechanism in NBCCS where the pathway is usually activated by haploinsufficiency for the patched-1 protein encoded by the PTCH1 gene [32, 41].

A non-recurrent deletion on chromosome 9q22.3 comprising a region of 352 Kb, in which the PTCH1 gene is present, has been shown to cause NBCCS and developmental delay and/or intellectual disability, metopic craniosynostosis, obstructive hydrocephalus, pre- and postnatal macrosomia, and convulsions. Affected individuals are also at increased risk for Wilms’ tumor. The clinical spectrum of the 9q22.3 deletion is variable, and the clinical results are somewhat dependent on the size of the microdeletion. 9q22.3 microdeletion cannot be identified by routine genetic analysis, except with extremely large deletions [32, 40].

#### Differential diagnosis

If macrocephaly and other neonatal defects are present, the presence of Sotos syndrome, Beckwith-Wiedemann syndrome (BWS), and isolated hydrocephalus or megalencephaly should be considered:

- Sotos syndrome is an autosomal dominant disease in which over 95% of cases have a pathogenic de novo variant. It is characterized by three typical clinical signs: characteristic facial appearance, learning difficulties, and overgrowth (increase in height and head circumference ≥2 standard deviation (SD) above average). The main features of Sotos syndrome include behavioral problems, advanced bone age, cardiac anomalies, cranial anomalies assessable by nuclear magnetic resonance (NMR) and computed tomography (CT), joint hyperlaxity/pes planus, maternal preeclampsia, neonatal jaundice, neonatal hypotonia, kidney abnormalities, scoliosis, and seizures. The risk of sacrococcygeus teratoma and neuroblastoma is slightly increased. Diagnosis is established in a proband by identifying a heterozygous pathogenic variant of the nuclear receptor binding SET-domain 1 (NSD1) gene, located on chromosome 5q35.3 [42, 43]
- Beckwith-Wiedemann syndrome (BWS) is a growth disorder characterized by neonatal hypoglycemia, macrosomia, macroglossia, hemihyperplasia, omphalocele, embryonic tumors (e.g., Wilms’ tumor, hepatoblastoma, neuroblastoma, rhabdomyosarcoma), visceromegaly, cytomegaly, corticosarcoma kidney (e.g., medullary dysplasia, nephrocalcinosis, medullary sponge kidney or Cacchi Ricci disease, nephromegaly), and folds/pits in the ears. Macroglossia and macrosomia are usually present at birth but may have a postnatal onset. The growth rate slows down around the age of 7 to 8. Hemihyperplasia can affect segmental regions of the body or selected organs and tissues. A provisional diagnosis of BWS based on clinical evaluation can be confirmed by molecular/cytogenetic tests. BWS is associated with abnormal regulation of gene transcription in two gene domains imprinted on chromosome 11p15.5 [32, 44, 45]
- Isolated hydrocephalus or megalencephaly can be diagnosed by clinical examination, family history, and x-ray examinations [32]

If the initial clinical signs are multiple BCCs, clinical examination and radiographs should diagnose NBCCS. However, there are other inherited disorders with similar skin signs:

- Brooke-Spiegler syndrome (BSS), also known as CYLD cutaneous syndrome (CCS). It is an autosomal dominant genetic disease characterized by trichoepitheliomas, milia (present only in areas exposed to the sun), and cylindromas. It occurs in the second or third decade of life and is caused by mutations in the CYLD gene, located on chromosome 16q12-16q13 [32, 46]
- Bazex-Dupré-Christol syndrome (BDCS). It is a dominant genetic disorder linked to chromosome Xq24-Xq27.1 caused by a gene mutation in the actin-related protein-testis 1 (ARP-T1/ACTRT1) gene, characterized by multiple BCCs, follicular atrophy on the back of the hands and feet (resembles the skin and is different from the palm-plantar pits of the NBCCS), hypohidrosis, and hypotrichosis [47, 48]
- Rombo syndrome. It is a dominant hereditary disease like Bazex-Dupré-Christol syndrome, reported in a single family. Skin signs are vermiculate atrophoderma, milia, hypotrichosis, trichoepithelioma, BCC, and peripheral vasodilation with cyanosis. The skin is normal until late childhood, BCCs develop in adulthood, and hypohidrosis is absent [49, 50]
- Autosomal dominant or X-linked syndrome with hypotrichosis and BCC, reported in a single family [51]
- Autosomal dominant inheritance of multiple BCCs in the absence of other characteristics [32]

If there is an exposure to arsenic, this can also be a differentiation point [52].

#### Radiological investigations

Gorlin-Goltz syndrome is characterized by a triad of typical manifestations: multiple basal cell nevi, odontogenic keratocysts, and skeletal deformities.

Radiological investigations play a fundamental role in the diagnosis of GGS. About 60% of patients have characteristic dysmorphisms such as macrocephaly, bulging forehead, and facial milia. Skeletal anomalies such as fused or cuneiform vertebrae, hemivertebrae, and kyphoscoliosis may be present, and there is a presence of facial dysmorphisms such as cleft lip/palate, macrocephaly, and ocular anomalies. The maxilla may appear hypoplastic and mandibular hyperplasia with variable prognathism may be present. Other less frequent skeletal anomalies are malocclusion and dental crowding, caused by the presence of keratocysts that can cause dislocation of the dental elements, non-eruption, and root resorption.

In a case report published by Nilesh et al. in 2017 [53], the usefulness of the radiographic investigation using orthopantomography was highlighted, as there were multiple radiolucent lesions present in both maxillary bones, some of which associated with the dental apexes of the third molars. Furthermore, the lesions present at the alveolar process caused thinning of the bone thickness.

The typical radiographic findings in the jaws are in fact multilocular radiolucent lesions, with well-defined sclerotic edges. Keratocysts are asymptomatic and often occasional findings, a factor that can explain the late diagnosis in some cases, but can become symptomatic in the event of infection, nerve compression, tooth mobility, or edema. They are more localized in the area of the mandibular branch, while the mandibular body and the upper jaw are less frequently affected areas. Keratocysts are usually surrounded by a few satellite cysts. This feature associated with high mitosis of the epithelium in the presence of a thicker fibrous capsule favors a high recurrence of the lesion after surgical removal; therefore, follow-up with radiographic examination is indicated. Other typical lesions can be found in radiographic images of the skull, where bilamellar calcification of the falx cerebri and alterations of the sella turcica are often highlighted.

### Part 2 — Treatment at levels of jawbones

The treatment of GGS is usually based on the surgical excision of the odontogenic keratocysts associated with the disease [54–56].

**Fig. 2:**
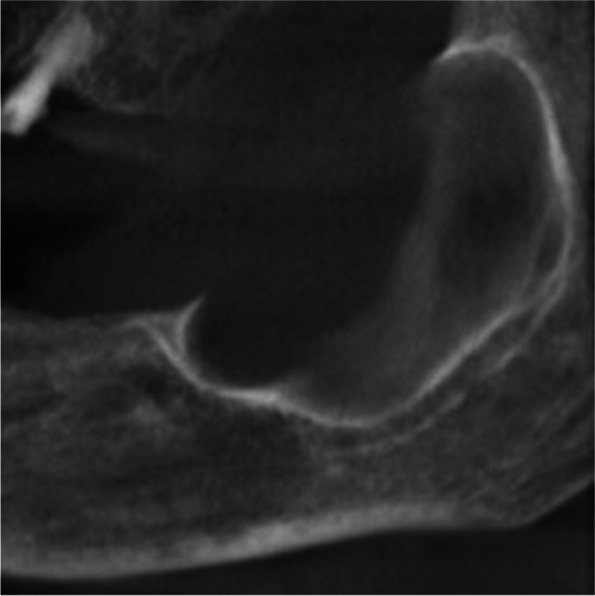
Sagittal view: wide unilocular oval cystic lesion of the left retromolar trigone. The lesion measures 40 mm and shows a sclerotic margin. The anterosuperior cortex is resorbed. The mandibular canal is separated from the lesion by a thin bone layer

**Fig. 3:**
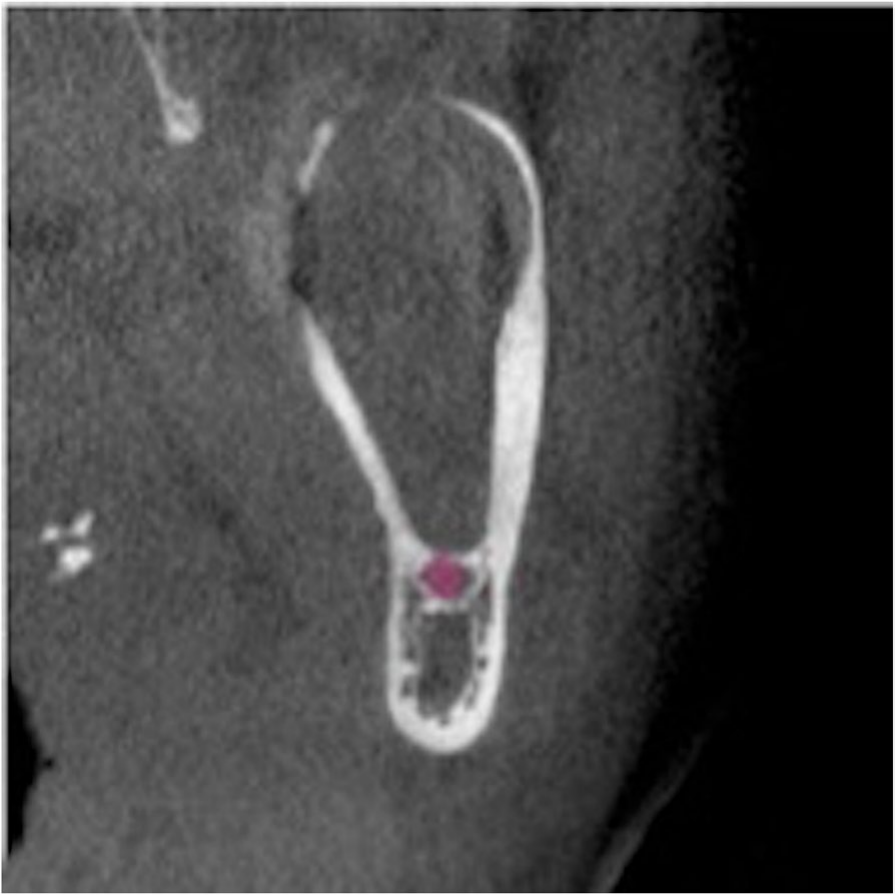
Coronal view

**Fig. 4:**
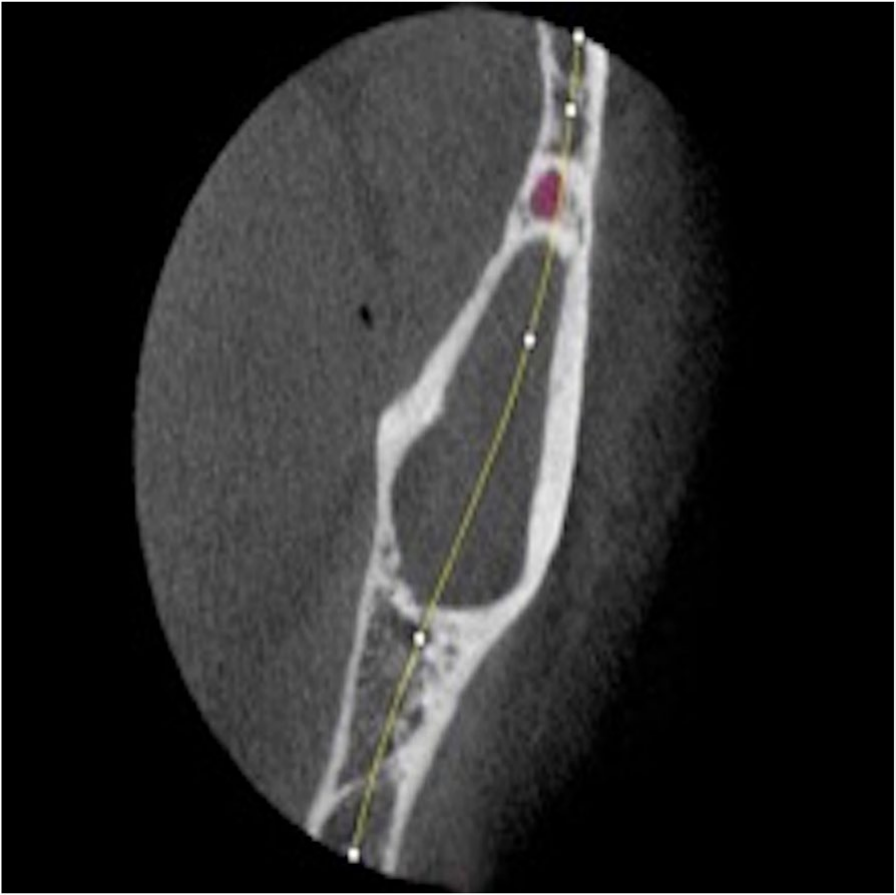
Axial view

**Fig. 5:**
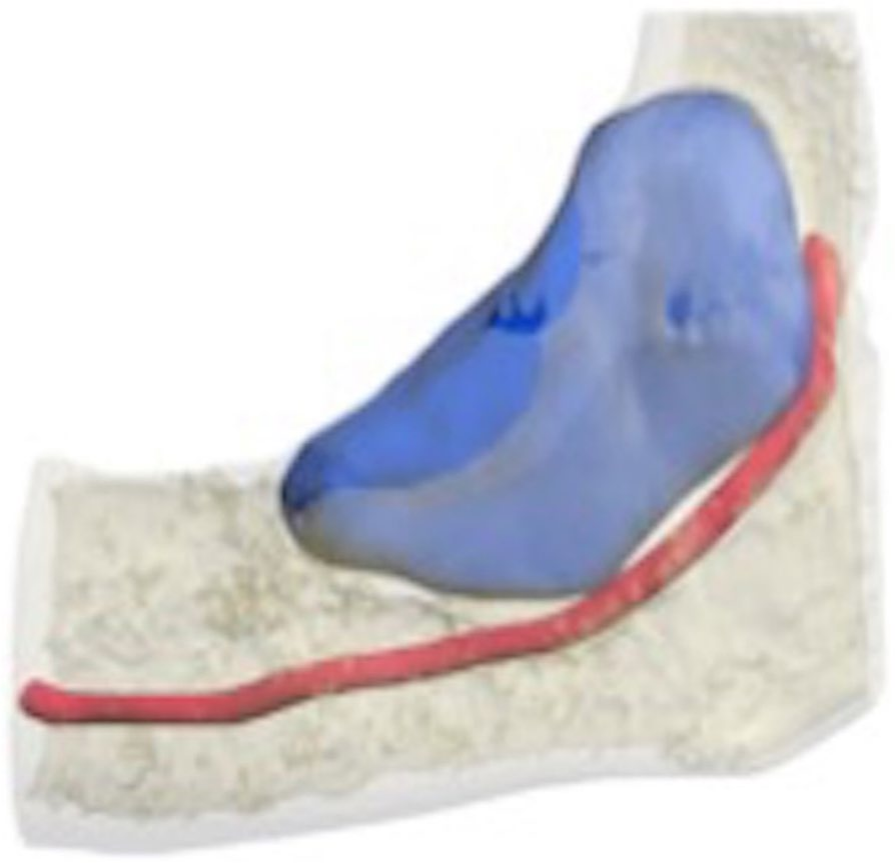
3D reconstruction of the keratocyst

There is a difference between odontogenic keratocysts associated with BCNS and not since keratocysts of odontogenic origin are generally single and found in adult and elderly patients, with a preference for the posterior portion of the mandible with thick epithelium and with a recurrence of about 61% (Figs. 2, 3, 4, and 5). The keratocysts associated with BCNS, on the other hand, appear multiple, in young patients, with an equal distribution in the jawbones, thinned lining epithelium, and have a recurrence of 82% of relapses [31, 32].

The potential recurrence of keratocysts treated with enucleation has guided the treatments on the choice of resective surgery, but recently, the preferred treatment plan is the intermediate one between enucleation and resective surgery. More extensive lesions preclude the possibility of using resection.

The recurrence rates for odontogenic keratocysts are variable: 30% following enucleation, 9–17% following enucleation and irrigation with Carnoy’s solution, 14.5–38% with enucleation and cryotherapy, 18% with enucleation and ostectomy peripheral, 13–14.6% with decompression and cystectomy, 32–33% with marsupialization, and 0–8.4% following surgical resection [54].

The causes of recurrence of keratocysts that have been hypothesized are tendency to multiplicity, fragility of the tissue lining which often precludes complete removal of the cyst, fragmented enucleation, intrinsic potential for epithelial replication, incomplete removal of the lining mucosa, retention of cyst satellites, and the complexity of complete removal of cysts present in the root zone of the dental elements. Keratocysts have a higher recurrence potential in BCNS and have onset at a young age; the cysts are generally parakeratinized and occasionally may have a neoplastic degeneration, giving rise for example to ameloblastomas and squamous cell carcinomas. For this reason, surgical treatment and careful follow-up are indicated [57, 58].

The current orientation provides — based on the histological type, localization, and type of development of the lesion — the following surgical protocols:

- In case of unilocular lesions that develop in the context of the mandible and that do not cross over into the surrounding soft tissues, a first approach is represented by the removal of the lesion associated with an energetic bone curettage with manual and rotating instruments, and the application of substances cytotoxic.
- In case of multilocular lesions that always develop in the context of the mandible, the treatment modality described above can still be adopted, but exposes to a greater risk of recurrence: if this occurs, a more radical approach will probably have to be used.
- In case of multilocular lesions that develop in the mandible and that have eroded the bone corticals with “encroachment” in the soft tissues, removal and curettage hardly offer the possibility of being radical. In these cases, the treatment may require “en bloc” resection with wide safety margins of the lesion already in the first instance, followed by immediate or deferred reconstruction of the portion of tissue removed.
- As regards the maxilla, benign but locally aggressive lesions must be treated from the beginning in a more radical way than mandibular ones, with the same extension, morphology, and histological type, due to the local anatomy (thin bone and quickly erodable, pneumatized spaces that offer poor resistance to tumor growth, greater proximity to the skull base, infratemporal fossa, etc.) which favors a more rapid development and which makes the treatment more complex in case of relapse [59–64].

Typical treatment schemes for odontogenic lesions that develop in the context of the jawbones are:

- Simple enucleation
- Enucleation with bone curettage in association or not with topical use of cytotoxic chemicals
- “En bloc” resection followed by possible bone reconstruction
- Marsupialization
- Decompression

#### Surgical treatment — enucleation

It consists in the complete removal of the cyst in a single operating session. The bone cavity residual typically undergoes spontaneous healing, with bone regeneration thanks to a mechanism of organization of the primitive blood clot that forms in the postoperative period. This method is advantageous in terms of timing, as it allows the resolution of the pathology in a single session and with faster healing times.

In the case of larger keratotic tumors that have already reached the periosteal plane and created dislocation or erosion of the bone cortices, complete excision is difficult due to the adhesions of its epithelium to the periosteal or mucous tissue. The attempt to preserve the adjacent dental elements or neurovascular structures and the difficulty of access in some sites can add up to the attempt to limit the extension of the surgical field and lead to incomplete removal, for example in the posterior sectors of the maxillaries and at the mandibular branch level. In cases of encroachment outside the periosteal capsule, the overlying mucosa, which merges with the cystic wall, must be carefully removed because it is one of the main sources of recurrence; in case of contiguity with dental elements, the most suitable treatment modality must be evaluated in order not to leave residues in their proximity (for example, extraction or root canal therapy and apicoectomy).

There are different types of enucleation:

- Simple enucleation: refers to the removal of the lesion from the bone in its entirety, without leaving macroscopic residues
- Enucleation with Carnoy’s solution: refers to the removal of the lesion, followed by the application of Carnoy’s solution within the surgical access. Carnoy’s solution (a mixture of 60% ethanol, 30% chloroform, glacial acetic acid, and ferric chloride) is used on the bone walls after surgical enucleation or in the cystic cavity before its removal; it is a cauterizing solution which, without penetrating deeply, seems to be able to eliminate any epithelial residue. Application should be avoided in correspondence with neurovascular bundles; in polycystic cavities, the bone septae must be previously eliminated to allow a correct action of the liquid [65].
- Enucleation with peripheral ostectomy: enucleation of the lesion is followed by a peripheral ostectomy. This technique is suggested as an additional approach to avoid surgical resection. The method includes the use of rotary instruments for the removal of a greater amount of bone, ensuring the elimination of all the residual lining epithelium. In the studies carried out, there is no evidence of recurrence of the lesion following this treatment
- Enucleation with peripheral ostectomy and application of Carnoy’s solution: enucleation of the lesion is followed by ostectomy and application of Carnoy’s solution inside the cavity. This approach shows very encouraging results, as no relapses have been highlighted, because the combination of the ostectomy and the application of Carnoy’s solution allows for good removal of epithelial residues and satellite cysts. Morbidity is significantly less than with resective surgery [66–68].

#### Surgical treatment — resection

Surgical resection or “en bloc” resection consists in the surgical removal of an entire section of the maxillary or mandibular bone without maintaining continuity. This demolition technique is the only approach that does not show recurrence of keratocysts in surgical follow-ups but, despite the high long-term success rate, it also produces a very high cost in terms of morbidity, such as the loss of bone continuity and mandibular and facial deformation. The resection can also be only marginal, which is an advantageous technique because it allows the lesions to be surgically removed, while maintaining an intact bone margin to ensure continuity. Resective treatment does not appear justified for asymptomatic lesions, even large ones, involving only the spongy part of the bone, without expansion, erosion, or perforation of the cortical bone. Resection should be reserved only for tumors that have perforated the cortical layer, especially those that are recurrent. The lesions limited to the medullary part, on the other hand, should generally be approached in a more conservative way. In the case of “en bloc” resection at the mandibular level, it is indicated, if possible, to proceed with the immediate reconstruction of the mandible with autologous bone grafts or free flaps of the fibula or iliac wing. In the case of extensive, multilocular lesions, in sites at risk or relapsing, in the phase of malignant degeneration or trespassing in the deep planes, the use of a more radical therapy, such as “en-bloc” resection, would seem more suitable for the purpose of avoid a recurrence of the lesion and its further expansion.

#### Surgical treatment — marsupialization

Marsupialization is the most conservative method of treating keratocystic lesion: the principle on which it is based is to make the cyst communicate widely with the oral cavity with consequent elimination of endocystic pressure. The drop-in blood pressure will cause a block osteoclastic activity and stimulation of repair with activation of osteoblasts, with a progressive reduction in the size of the lesion.

It has two main indications:

- Cases in which enucleation exposes to significant intraoperative risks
- Follicular cysts which contain important dental elements

The advantages are represented by the simplicity of execution (possibility of treatment under anesthesia also of large lesions), by the reduced risk of iatrogenic fractures and of neurovascular lesions, and by the elimination of the risk of loss of vitality of dental elements plunging into the lesion.

The progressive decrease of the cystic size over time allows to safeguard the surrounding anatomical structures (maxillary sinus, dental elements, neurovascular bundles), to minimize the risk of fracture and to carry out the subsequent enucleation in the presence of a lesion that is technically easier to remove.

The disadvantages of marsupialization are represented by a resolution of the pathology very slow with discomfort for patients, as an accessory cavity is created in the oral cavity difficult to cleanse.

Furthermore, this method requires a great collaboration on the part of the patient who must undergo irrigations of the residual surgical communication and frequent follow-up.

It has been observed at histological level that, in the biopsy material taken from the healed surface of the surgical access after the resolution of the lesion, there are no signs of epithelial residues of the cyst or daughter cysts. In the studies that have been conducted, in the biopsies prior to surgery, the presence of the expression of B-cell lymphoma 2 (bcl-2) in the cystic epithelium, but not in the material taken after the procedure, is noted. Furthermore, there is also a reduced expression of interleukin-1 alpha (IL-1α). These two factors have been associated with the expansion of the lesion [69–71].

#### Surgical treatment — decompression

Decompression is a technique very often confused with marsupialization, but with a different approach. In fact, this method involves the opening of the cystic lesion through a small incision and the insertion of a catheter to maintain the opening and drainage. Decompression is often followed by cystectomy a few months later, when the size of the lesion has reduced. In this way, the second step of the surgical technique can be performed without damaging important anatomical structures, such as the teeth or the inferior alveolar nerve, also reducing the possibility of spontaneous fracture.

The reason for decompression is to reduce the pressure inside the cystic lesion, an objective also achieved through marsupialization, to expose the capsule of the lesion to the oral environment for a definitive resolution. Both methods, decompression and marsupialization, are particularly effective in pediatric treatments and in patients with severe pathologies and extensive lesions. The advantage of these techniques is that they are minimally invasive, can be performed under local anesthesia, and avoid the risk of resection and consequent deformity of the face.

Both decompression and marsupialization, although considered effective techniques, have cases of relapse in long-term follow-up [69, 70].

#### Surgical treatment — cryotherapy

Cryotherapy consists in the use of a nitric oxide cryoprobe at a temperature of about −20 ° C or lower on the bone walls after enucleation or on the soft tissues if the lesion has perforated the bone plane and encroaches beyond the periosteal capsule. The application lasts 1 min and is repeated twice with an interval of 5 min. Cryotherapy appears to be able to produce cellular necrosis at the bone level, but to keep the inorganic component intact, maintain the extracellular architecture, and facilitate new bone formation; cell death occurs due to direct damage caused by the formation of intra- and extracellular ice crystals, osmotic and electrolytic disturbances. This method allows a relative absence of bleeding and scarring; however, due to the difficulty in controlling the amount of liquid nitrogen applied to the cavity, the resulting necrosis and swelling can be unpredictable. In addition, an increased risk of subsequent spontaneous fracture was also observed [66].

## Conclusions

Gorlin-Goltz syndrome is an autosomal dominant genetic process, except for sporadic cases of the mutation, which is of particular interest to oral maxillofacial health experts. In order to arrive at an early diagnosis of the syndrome, it would be advisable to perform radiographic and clinical tests from the young age of the patient. Treatment therefore requires a multidisciplinary approach between different health areas to ensure an adequate quality of life and effective treatment of the symptoms.

As for the surgical treatment of cystic lesions typical of the pathology at the level of the jawbones, there is currently no treatment of choice for resolution. In fact, based on the extent of the keratocysts, the histology, the location and characteristics of the patient, and the advantages and disadvantages related to each surgical approach must be evaluated. As for the relapse rate associated with the use of one technique over another, literature reports OKC is a typical developmental cyst with a recurrence rate of 12 to 58.3%. The reasons for this wide range could be difference in the number of cases and the duration of observation, whether lesions with ortho- or parakeratinized epithelium were included, and whether lesions associated with basal nevus syndrome were included. Although the exact reason for the high recurrence rate of OKC has not been established, it is thought to be due to incomplete removal of the primary lesion [69, 72].

An appropriate long-term follow-up must be done after surgical treatment performed in order to ensure clinical success, which means an absence of signs of recurrent disease. Some literatures report especially odontogenic keratocyst recurred more than 10 years after enucleation, which means that odontogenic keratocyst must be followed up for more than a decade. The minimal follow-up period after surgery of OKC could be for children 6 months and adult 1 year. The observation period of recurrence in literature varies from 1 to 10 years [73].

The follow-up period is established as the interval between the surgical treatment and the most recent consultation, which consists of clinical and radiographic evaluations (panoramic radiographs for all of the cases and CT scans for cases in which an anatomic superimposition to other structures compromises a simple radiographic evaluation) and confirmation by histopathological diagnosis of OKC [74–76].

The follow-up should involve performing an orthopantomography every 6 months in young patients and a CBCT in case of doubt, or to evaluate contiguity with anatomical structures such as neurovascular bundles, teeth, and maxillary sinuses [75, 76]. For odontogenic keratocysts in adults and elderly patients, the follow-up seems to be longer [77].

## Data Availability

Not applicable

## Abbreviations

ARP-T1/ACTRT1: Actin-related protein-testis 1
BCC: Basal cell carcinoma
Bcl-2: B-cell lymphoma 2
BCNS: Basal cell nevus syndrome
BDCS: Bazex-Dupré-Christol syndrome
BSS: Brooke-Spiegler syndrome
BWS: Beckwith-Wiedemann syndrome
CBCT: Cone beam computed tomography
CCS: CYLD cutaneous syndrome
CT: Computed tomography
CYLD: Cylindromatosis
DHH: Desert hedgehog
GGS: Gorlin-Goltz syndrome
GLI: Glioma-associated oncogene
GLI1: Glioma-associated oncogene homolog 1
GLI2: Glioma-associated oncogene homolog 2
GLI3: Glioma-associated oncogene homolog 3
GLIA: Glioma-associated oncogene activator
GLIR: Glioma-associated oncogene repressor
Hh: Hedgehog
IHH: Indian hedgehog
IL-1α: Interleukin-1 alpha
MC1R: Melanocortin 1 receptor
NBCCS: Nevoid basal cell carcinoma syndrome
NMR: Nuclear magnetic resonance
NSD1: Nuclear receptor binding set-domain 1
OKC: Odontogenic keratocyst
PTC: Patched
PTCH1: Protein patched homolog 1
PTCH2: Protein patched homolog 2
RNA: Ribonucleic acid
SHH: Sonic hedgehog
SMO: Smoothened
SSD: Sterol-sensing domain
SUFU: Suppressor of fused homolog
